# Supplementary material for: Simultaneous Analysis of Hydroquinone, Arbutin, and Ascorbyl Glucoside Using a Nanocomposite of Ag@AgCl Nanoparticles, Ag2S Nanoparticles, Multiwall Carbon Nanotubes, and Chitosan
Source: Nanomaterials (Basel). 2020 Aug 12;10(8):1583. doi: 10.3390/nano10081583 (PMC7466507; doi:10.3390/nano10081583)
Supplement: Supplementary file 1 [file nanomaterials-10-01583-s001.pdf]

nanomaterials

MDPI

Article

Simultaneous Analysis of Hydroquinone, Arbutin, and Ascorbyl Glucoside Using a Nanocomposite of Ag@AgCl Nanoparticles, Ag_2S Nanoparticles, Multiwall Carbon Nanotubes, and Chitosan

Nutthaya Butwong [1,*], Thidarat Kunawong [1] and John H.T. Luong [2]

1 Applied Chemistry Department, Faculty of Sciences and Liberal Arts, Rajamangala University of Technology Isan, 744, Suranarai Rd., Nakhon Ratchasima 30000, Thailand; thidarat.ku@gmail.com
2 School of Chemistry, University College Cork, Cork, T12 YN60, Ireland; luongprof@gmail.com
* Correspondence: nbutwong@gmail.com

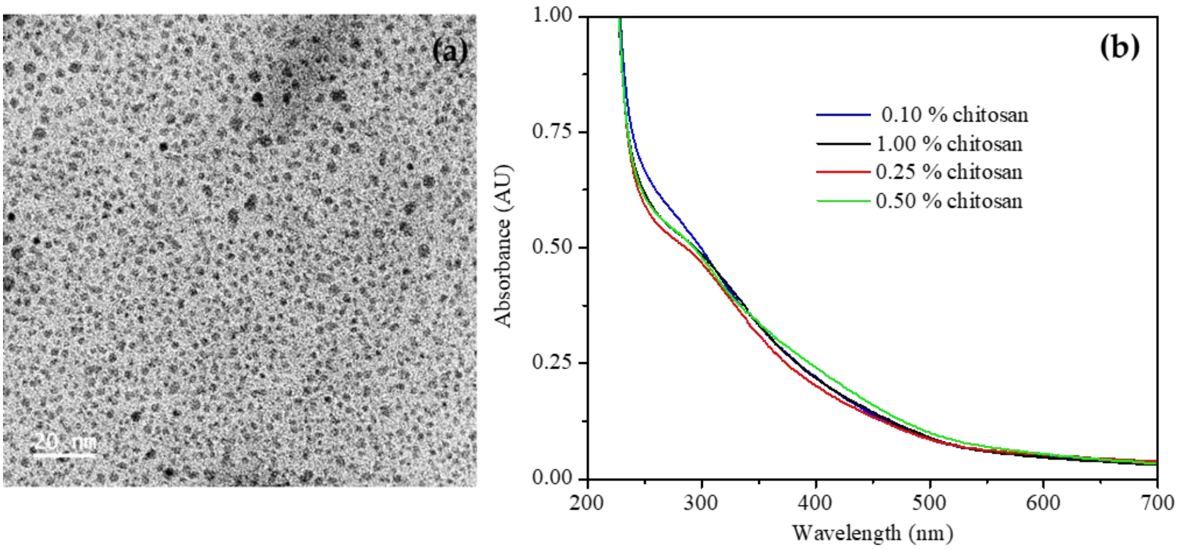

Figure S1. (**a**) A representative TEM micrograph of the synthesized Ag_2S nanoparticles (diluted 20 times by deionized water) and (**b**) UV-vis absorption spectra of Ag_2S using different concentrations of chitosan for the synthesis.

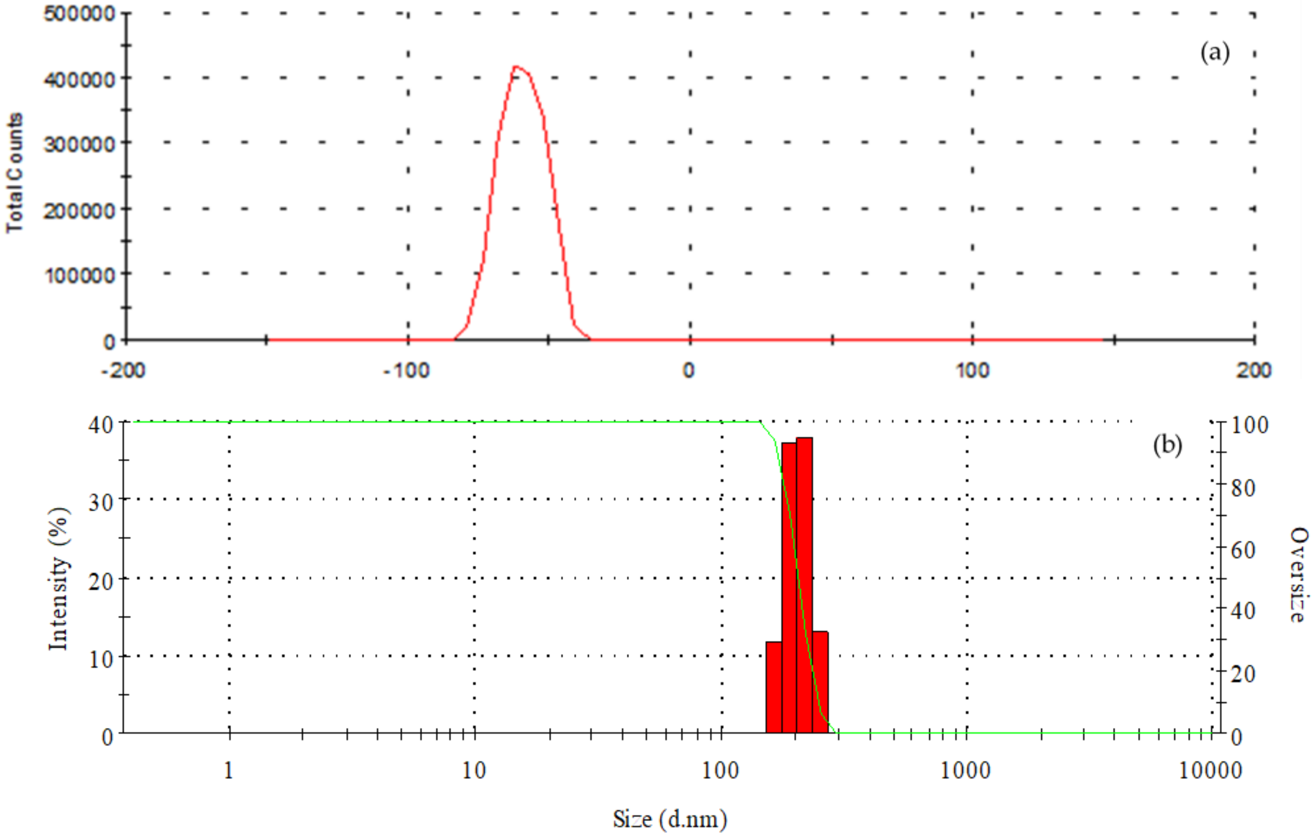

Figure S2. The zeta potential of Ag_2S nanoparticles stabilized by chitosan (**a**) and their relative size distribution (**b**).

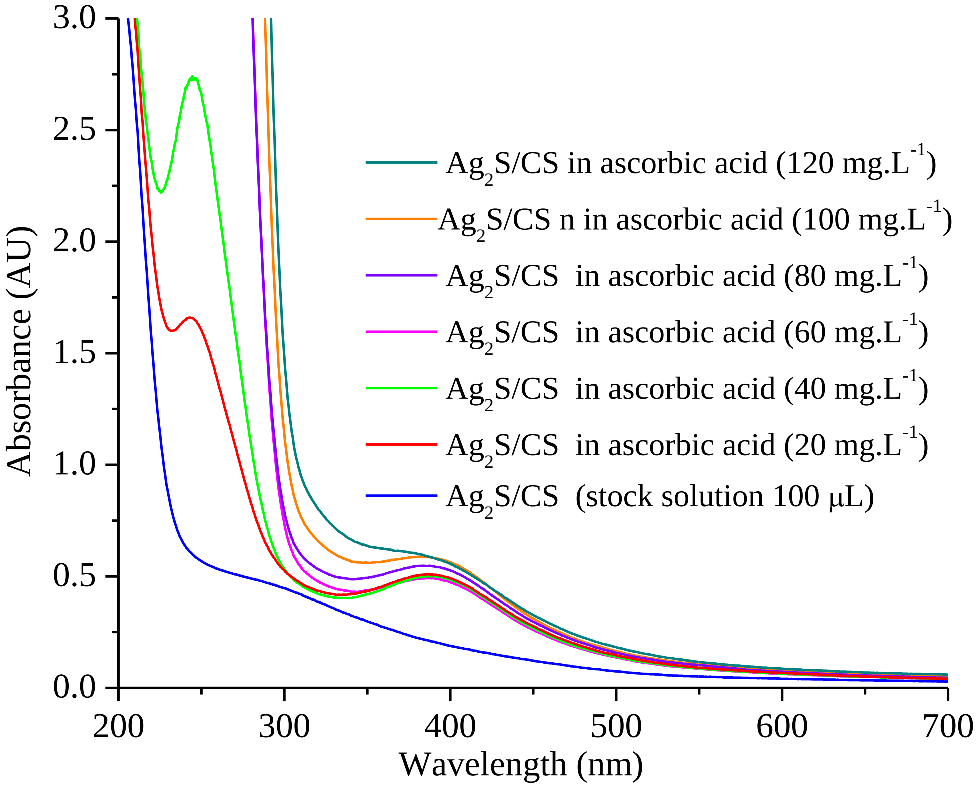

Figure S3. The absorption spectra of Ag_2S (10 times dilution by DI water) in the presence of ascorbic acid at different concentrations.

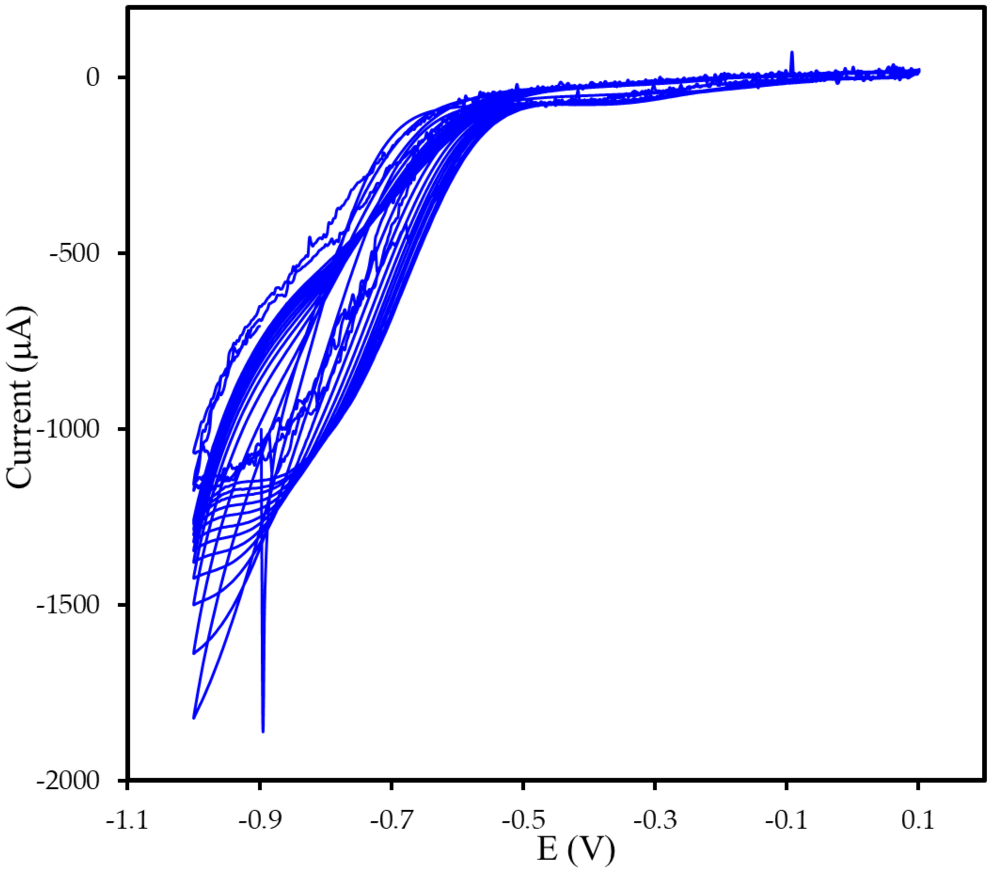

Figure S4. Cyclic voltammograms (CVs) with 15 cycles of the CNTs/GCE in the colloidal Ag_2S-chitosan mixture at a scan rate of 20 $mV.s^{-1}$.

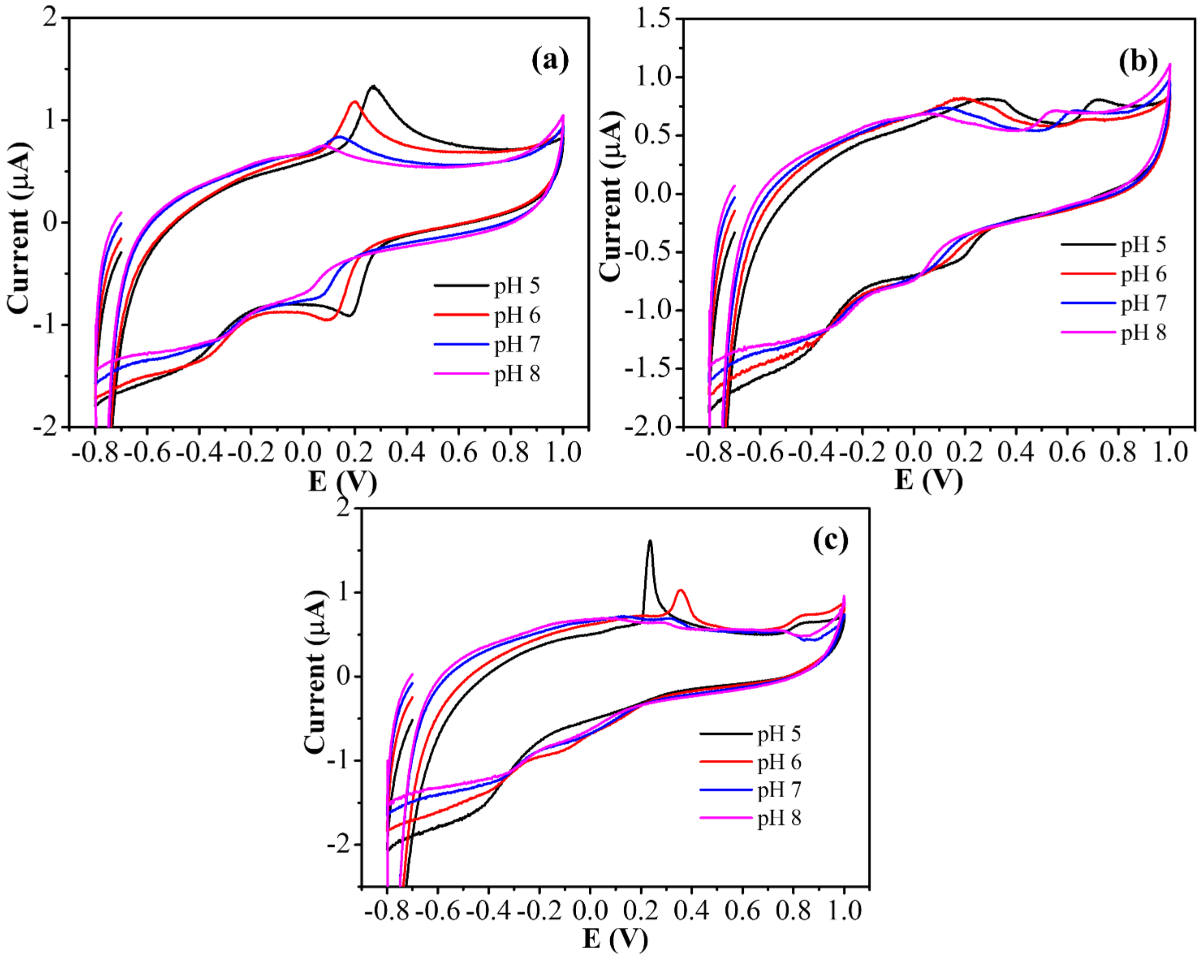

Figure S5. The CVs of HQ 7.1 μM (**a**) AR 4.0 μM (**b**) and AA2G 3.1 μM (**c**) in 0.1 M phosphate buffer at different pHs (5.0–8.0).

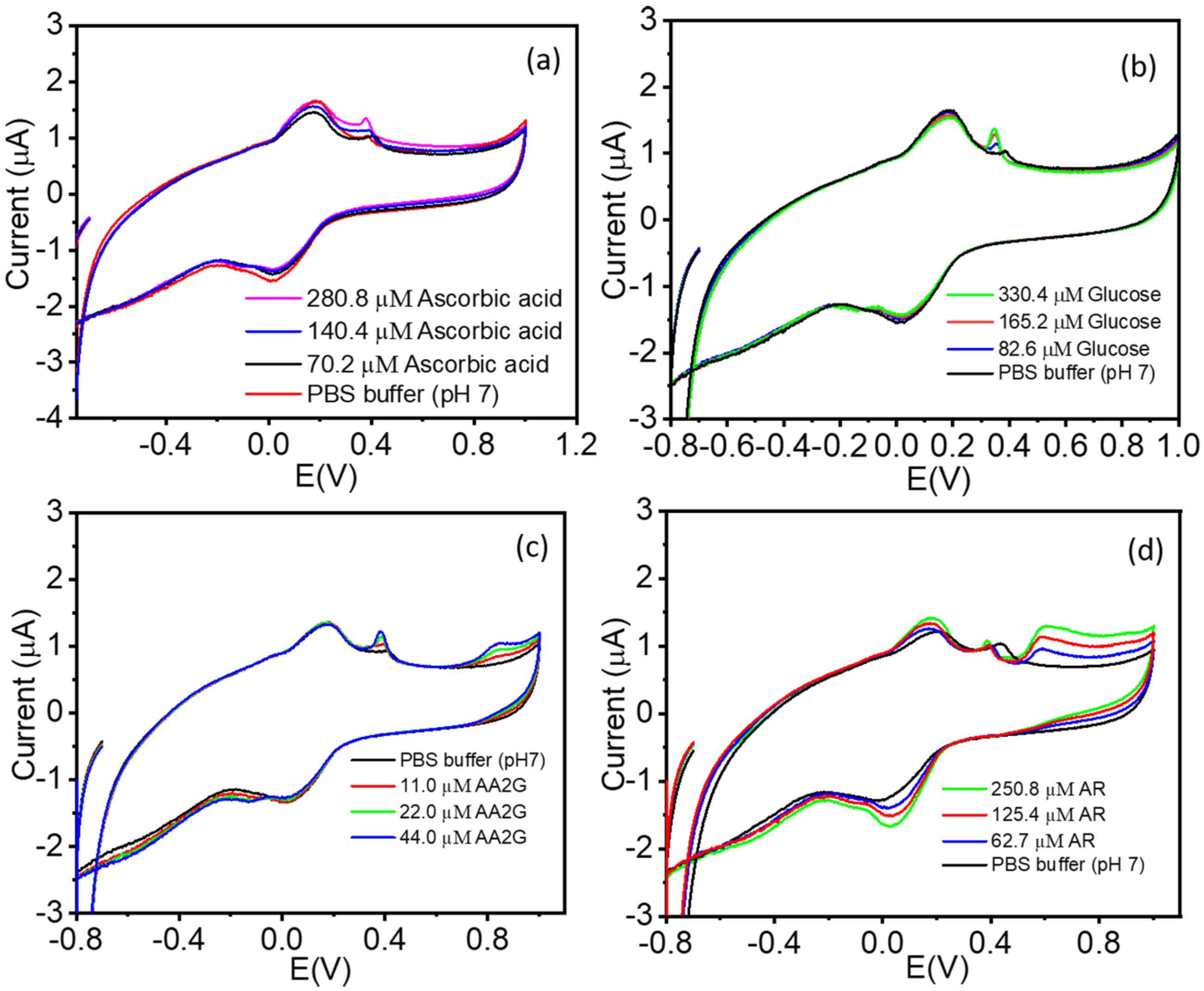

Figure S6. The CVs of ascorbic acid (**a**) glucose (**b**) AA2G (**c**) and AR (**d**) at different concentrations on the Ag@AgCl/Ag_2S/CNTs/GCE in 0.1 M phosphate buffer, pH 7.

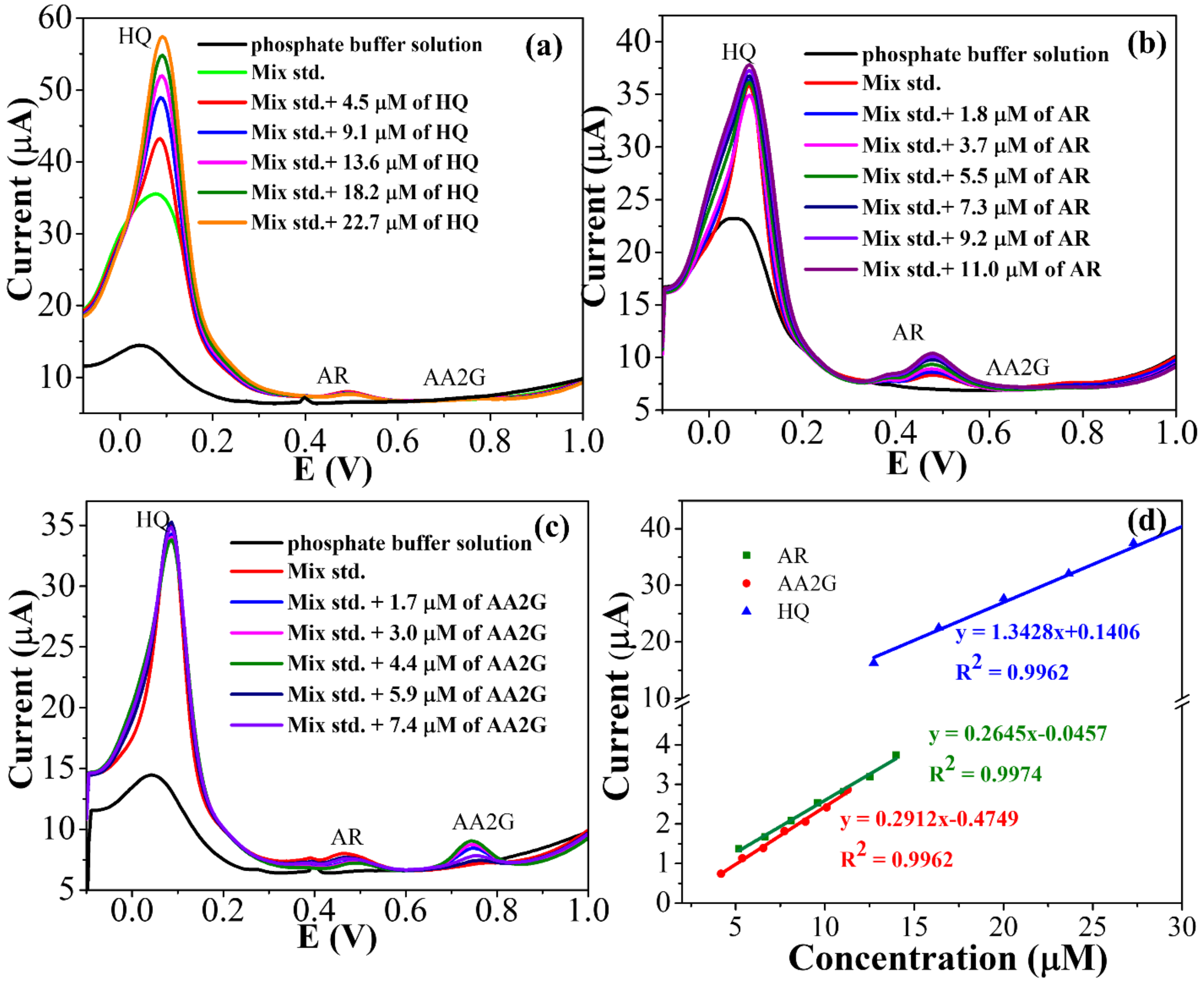

Figure S7. DPVs of the Ag@AgCl/Ag_2S/CNTs/GCE, (**a**) with different HQ concentrations, while AR = 3.7 μM and AA2G = 3.0 μM, (**b**) DPVs with different AR concentrations, while HQ = 9.1 μM and AA2G = 3.0 μM (**c**) DPVs with different AA2G concentrations while HQ = 9.1 μM and AR = 3.7 μM (**d**) Calibration plot of anodic current (μA) vs. concentration for each analyte (μM).

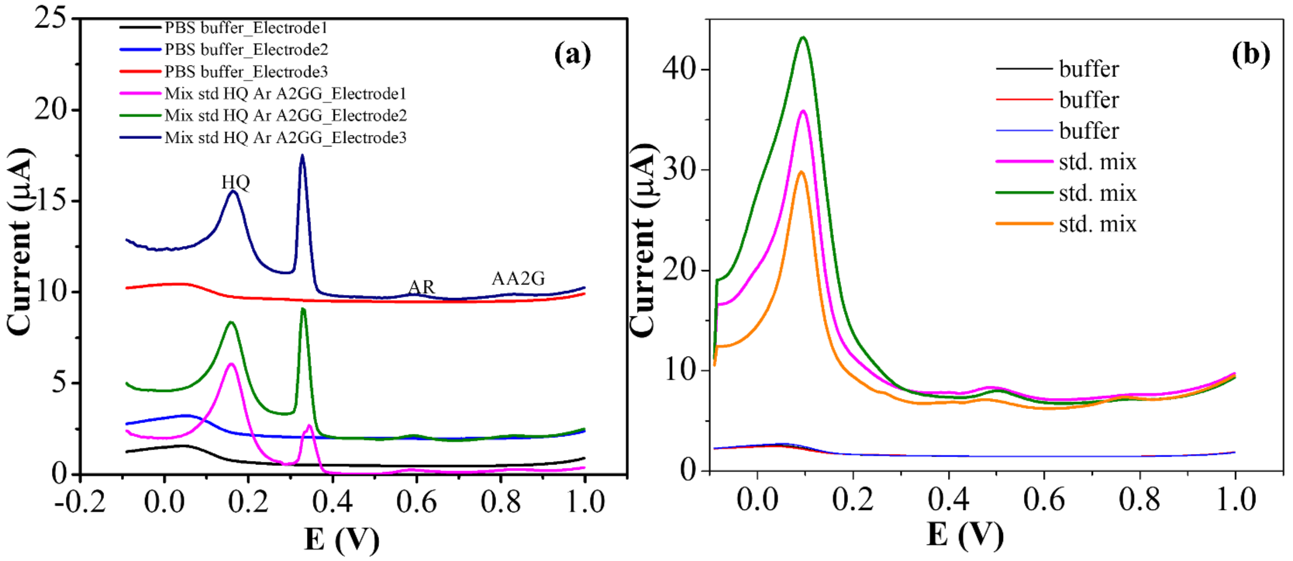

Figure S8. The DPV response obtained from the reproducibility of the Ag@AgCl/Ag_2S/CNTs/GCE (**a**) and the repeatability (**b**) studies of using the Ag@AgCl/Ag_2S/CNTs/GCE for the determination HQ (9.1 μM), AR (3.7 μM) and AA2G (3.0 μM).

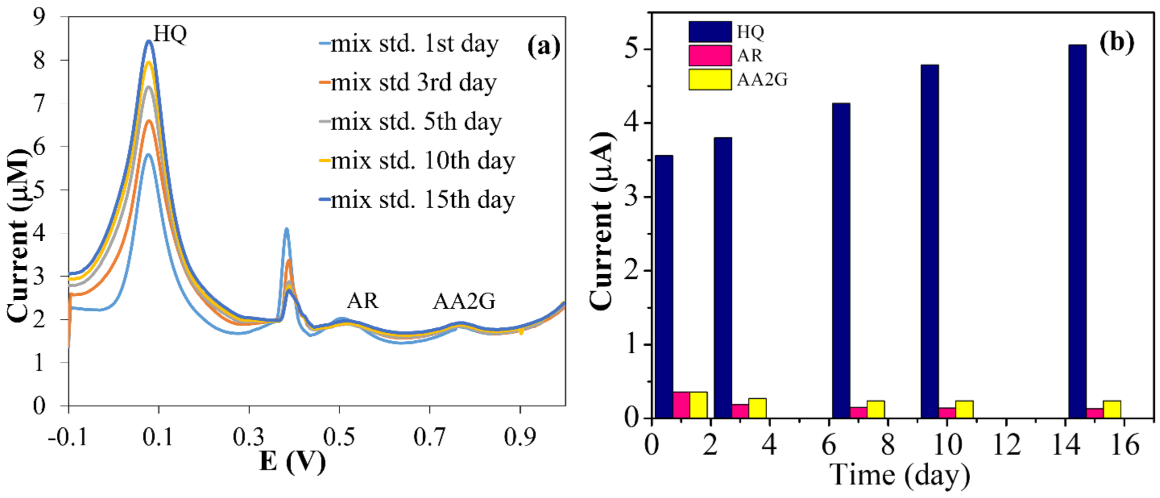

Figure S9. The CVs responses of using the same Ag@AgCl/Ag_2S/CNTs/GCE for determination of the analytes (40 ppb each) in a different day (**a**) and the anodic current of the analytes obtained by CVs in Figure S9a (**b**).

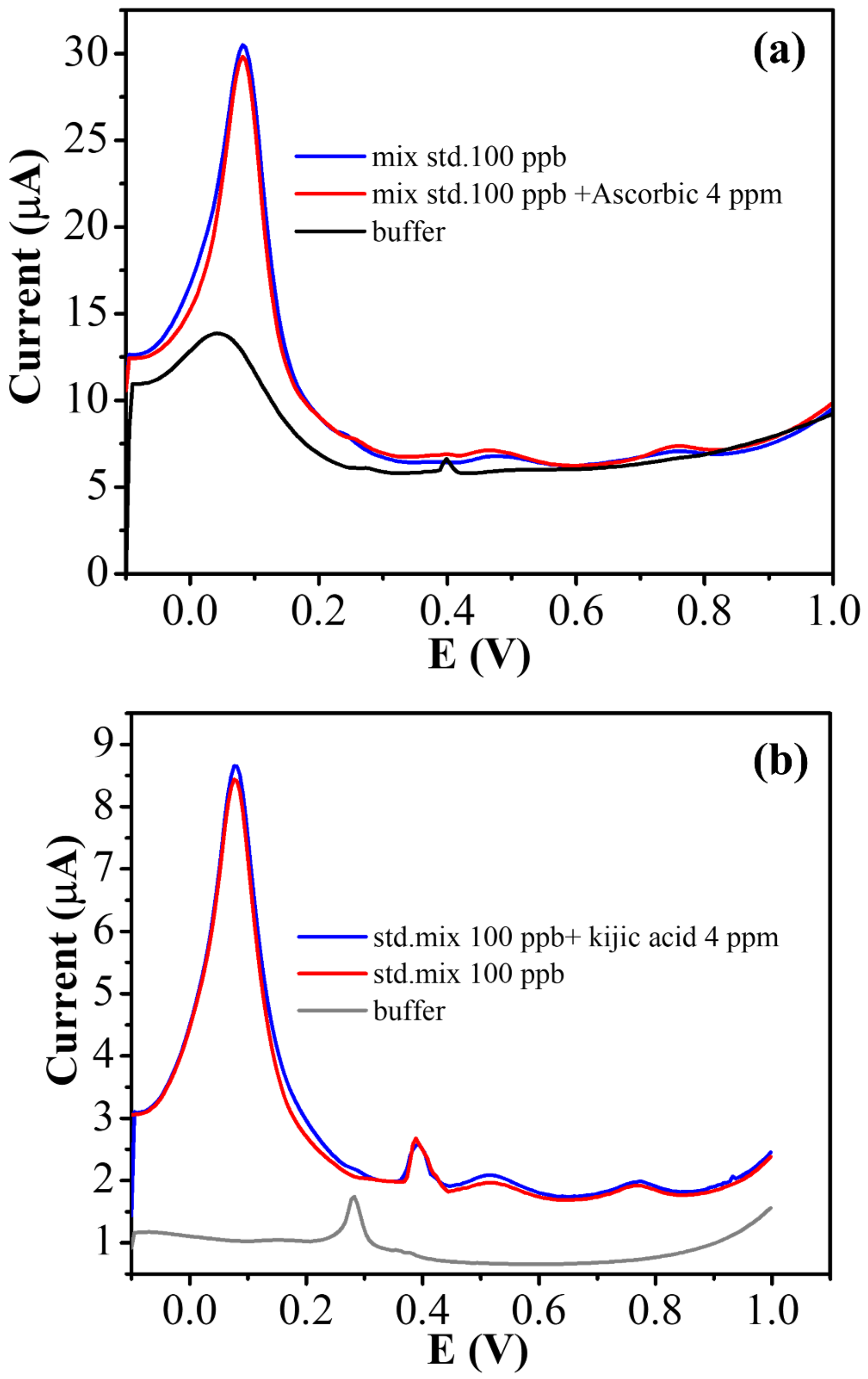

Figure S10. The effect of ascorbic acid (**a**) and kojic acid (**b**) on the response signal of the simultaneous determination HQ, AR, and AA2G using the Ag@AgCl/Ag_2S/CNTs/GCE.

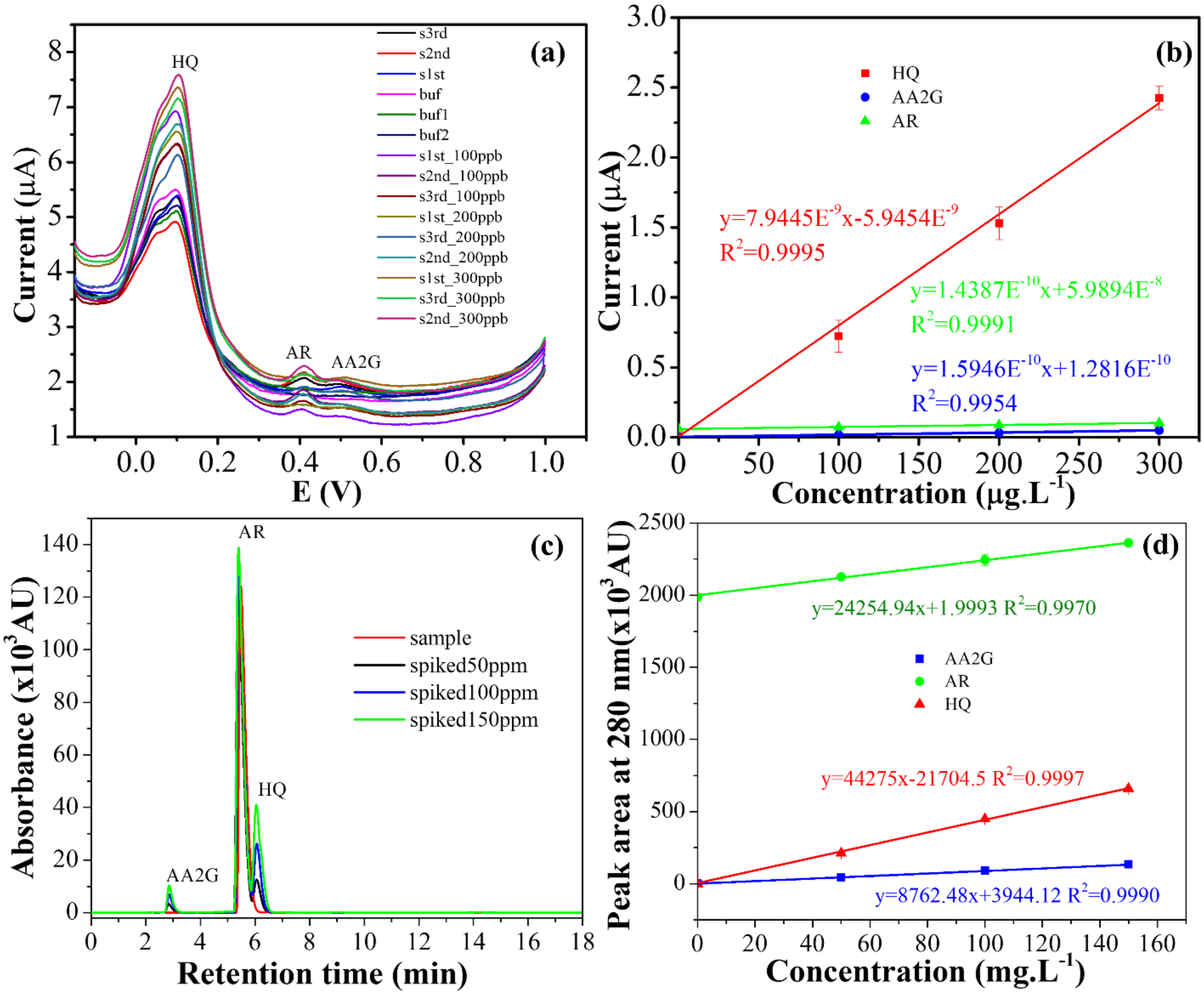

Figure S11. DPV responses of the Ag@AgCl/Ag_2S/CNTs/GCE when a mixture of HQ, AR, and AA2G (100, 200 and 300 μg·L^{-1}) was spiked in the whitening lotion (**a**) and the standard additions calibration curves (**b**) and chromatograms of the separation the spiked analytes sample (0, 100 and 150 mg·L^{-1}) on a C18 column (**c**) and standard addition calibration curves for HQ, AR, and AA2G (**d**) HPLC-PDA condition was XBridge C_{18} 3.5 um, 4.6 x 100 mm HPLC column using a mobile phase consisting of the MeOH:50 mM phosphate buffer (pH 2.5) at a ratio of 4:96 with a flow rate 0.5 mL.min^{-1}. All analyte absorbances were detected at 280 nm with an injection volume of 20 μL.

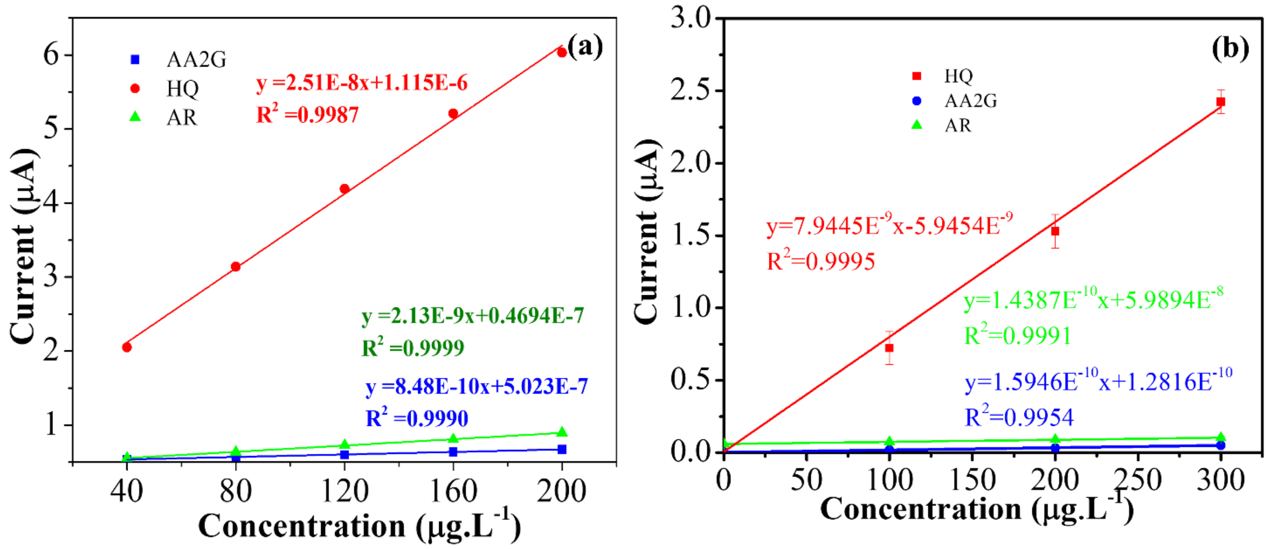

Figure S12. Calibration plots of the lotion-spiked standard analytes (**a**) and the calibration curves of the standard analytes (**b**).

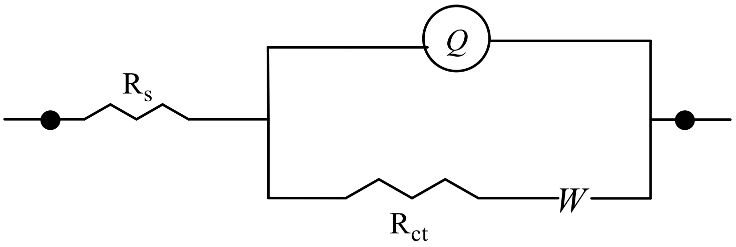

Figure S13. The modified Randles circuit is expressed as $R_s(Q(R_{ct}W))$: R_s (Ω) = resistance of the liquid electrolyte, CPE (constant phase element of pseudo-capacitance) = Q^n (Farad), n = 1, true capacitance, n = 0: pure resistance, W = Warburg elelement: $Z_W = A_W/(j\omega)^{0.5}$, where A_W is Warburg coefficient ($\Omega.s^{-0.5}$). The double layer capacitance (C_{dl}) is replaced by CPE.

The experimental data were fitted using ZSimpWin software to estimate the model parameters of the modified Randles circuit (Table S1).

Table S1. EIS parameters of the modified glassy carbon electrode in 0.1 M KCl containing 5 mM of $Fe(CN)_6^{4-/3-}$.

	GCE	CNTs	Ag_2S-CNTs	Ag@AgCl/Ag_2S-CNTs
R_s (Ω)	97.7	75.1	94	70
CPE (Q)	2.43×10^{-6}	7.44×10^{-4}	3.16×10^{-6}	6.06×10^{-6}
n	0.78	0.21	0.75	0.74
R_{ct} (Ω)	386.8	29.3	87.2	46.4
A_W ($\Omega.s^{-0.5}$)	0.00225	0.00227	0.0022	0.0027
Chi square	1.38×10^{-3}	1.047×10^{-3}	6.7×10^{-4}	4.59×10^{-4}

Table S2. The matrix effect of the lotion sample on the determination HQ, AR, and AA2G.

	The slope of the standard addition calibration curve (matrix)	The slope of the calibration curve (solvent)	%ME
	The proposed method (DPV)		
HQ	7.94×10^{-9}	2.51×10^{-8}	31.65
AR	1.44×10^{-10}	2.13×10^{-9}	6.75
AA2G	1.5946×10^{-10}	8.48×10^{-10}	18.75
	HPLC-PDA method (detect at 280 nm)		
HQ	4.43×10^{4}	1.71×10^{4}	259.34
AR	2.06×10^{4}	4.33×10^{4}	47.51
AA2G	9.05×10^{3}	9.30×10^{3}	97.32

© 2020 by the authors. Licensee MDPI, Basel, Switzerland. This article is an open access article distributed under the terms and conditions of the Creative Commons Attribution (CC BY) license (http://creativecommons.org/licenses/by/4.0/).
